# Reversible Cerebral Vasoconstriction Syndrome With Typical and Atypical Symptoms: A Case Series

**DOI:** 10.7759/cureus.55066

**Published:** 2024-02-27

**Authors:** Gayathri M Sivagurunathan, Dimitrios Fotopoulos

**Affiliations:** 1 Radiology, Indira Gandhi Medical College and Research Institute, Pondicherry, IND; 2 Radiology, University Hospitals of Morecambe Bay NHS Foundation Trust, Lancaster, GBR

**Keywords:** reversible cerebral vasoconstriction syndrome, thunderclap headache, mri, subarachnoid haemorrhage, primary angiitis of the central nervous system, ct, ct angio

## Abstract

Introduction

Reversible cerebral vasoconstriction syndrome (RCVS) is most commonly characterized by focal or diffuse severe thunderclap headache with or without focal neurological deficits and associated transient focal vasoconstriction of the intracerebral arteries lasting up to three months. We present six patients diagnosed as RCVS, with three patients presenting with focal neurological deficits without headache and the remaining three with severe headache alone. Neuroimaging revealed focal subarachnoid bleed with or without segmental intracerebral vasospasm, which resolved over three months. Despite thunderclap headache being the most prevalent symptom associated with RCVS, the absence of this symptom should not preclude the diagnosis, especially in the presence of cortical subarachnoid hemorrhage (SAH) or focal segmental intracerebral arterial narrowing.

Methods

This case series is a retrospective analysis of all patients diagnosed with RCVS between 2018 and 2022, focusing on clinical symptoms, imaging findings, and management.

Results

Six patients (three males and three females) were diagnosed with RCVS between 2018 and 2022. Three patients presented with typical symptoms, while the remaining three presented with atypical symptoms. Neuroimaging findings ranged from normal to focal SAH with or without arterial narrowing.

Conclusion

This case series underscores the diverse clinical presentations of RCVS, emphasizing that while thunderclap headache is the predominant symptom, its absence should not exclude the possibility of RCVS, especially when accompanied by focal neurological deficits or cortical SAH. Neuroimaging played a crucial role in identifying the spectrum of findings. These findings highlight the importance of comprehensive evaluation and consideration of RCVS in patients presenting with neurological symptoms, even in the absence of typical headache features.

## Introduction

Reversible cerebral vasoconstriction syndrome (RCVS) is a rare but intriguing neurological disorder characterized by the reversible constriction of cerebral arteries, leading to a spectrum of clinical manifestations. The hallmark of RCVS is the sudden onset of severe headache, often called thunderclap headache, accompanied by various neurological symptoms. While thunderclap headache is considered the classic presentation of RCVS, recent literature suggests that this syndrome can manifest without this typical feature, posing diagnostic challenges for clinicians [1].

The exact etiology remains unknown, with a suggestion of a transient disturbance in the cerebral vascular tone, which may be spontaneous, or after exposure to triggering agents such as sympathomimetic agents, peripartum period, or strenuous activities, resulting in cerebral vasoconstriction that resolves within a few months [2]. The radiological findings include focal subarachnoid hemorrhage (SAH), intraparenchymal hemorrhage, infarct, cerebral edema, or may be normal. Given the overlap in symptoms and radiological findings with other causes, it is crucial to distinguish RCVS from other etiologies, particularly vasculitis, to ensure appropriate management [3]. Despite thunderclap headache being the most prevalent symptom associated with RCVS, the absence of this symptom should not preclude the diagnosis, especially in the presence of cortical SAH or focal segmental intracerebral arterial narrowing [4].

This case series aims to shed light on the diverse clinical presentations of RCVS, exploring cases with the characteristic thunderclap headache and those without it. By delving into these varied presentations, we seek to enhance our understanding of the diagnostic nuances and management considerations associated with RCVS. Each case in this series serves as a unique clinical narrative, providing valuable insights into the spectrum of symptoms, radiological findings, and treatment responses observed in patients with RCVS.

Through a comprehensive examination of these cases, we hope to contribute to the existing knowledge surrounding RCVS and promote a nuanced approach to its diagnosis and management. The exploration of RCVS cases with and without typical thunderclap headaches not only highlights the heterogeneity of this syndrome, it also underscores the importance of a thorough clinical evaluation in capturing its diverse clinical phenotypes.

## Materials and methods

This case series is a retrospective analysis of patients who were treated for reversible vasoconstriction syndrome between 2018 and 2022 focusing on the clinical manifestations and neuroimaging findings associated with RCVS.

Six patients were diagnosed with RCVS during this period. Clinical data, including patient demographics, presenting symptoms, comorbidities, past medical history, laboratory investigations (including cerebrospinal fluid analysis), and details of the clinical course, were extracted from electronic medical records. Imaging studies, including computed tomography (CT) scans, magnetic resonance imaging (MRI), and angiography, were reviewed to assess the associated radiological findings. Follow-up imaging results were also analyzed.

## Results

Cases 1-3

**Figure 1:**
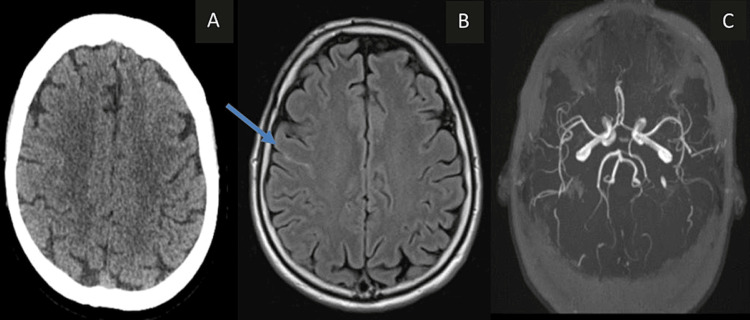
A 54-year-old man with sudden onset of left-sided paresthesia and facial numbness. (A) Unenhanced CT appears normal. There was no SAH. (B) FLAIR MRI on the same day shows focal SAH (blue arrow) in the right central sulcus. (C) MR angiogram was normal. Follow-up CT angiogram (not shown) was also normal CT: computed tomography; SAH: subarachnoid hemorrhage; FLAIR: fluid-attenuated inversion recovery; MRI: magnetic resonance imaging.

A 54-year-old man experienced a sudden onset of left-sided paresthesia and facial numbness, with no prior history of hypertension, diabetes, or any medications. Despite normal blood pressure on examination and an initial unremarkable non-contrast CT, a subsequent MRI revealed focal SAH in the right central sulcus (Figure 1). The initial CT and MRI angiograms displayed no abnormalities, and the cerebrospinal fluid (CSF) analysis was normal. However, a repeat CT angiogram after two weeks showed no focal abnormalities. The scan was repeated to ensure that there was no occult aneurysm particularly because the patient's symptoms had not completely resolved at that time. Symptoms gradually improved over four months without recurrence.

**Figure 2:**
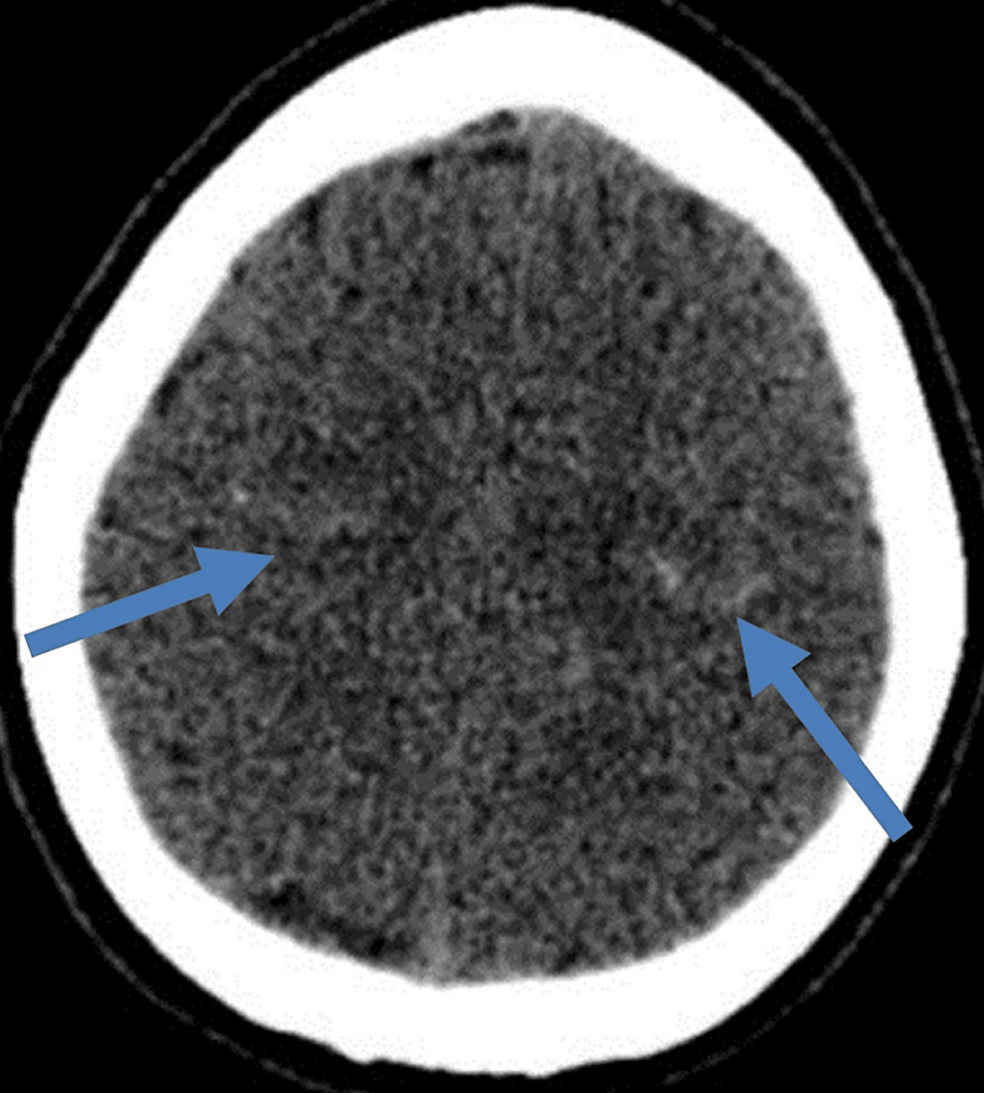
A 60-year-old man with a sudden, severe frontal headache accompanied by vomiting. Unenhanced CT shows focal SAH (blue arrows) in bilateral precentral sulci. CT angiogram (not shown) was normal. CT: computed tomography; SAH: subarachnoid hemorrhage

In the second case, a previously healthy 60-year-old man presented with a sudden, severe frontal headache accompanied by vomiting, with no apparent precipitating factors or neurological deficits. The initial CT scan revealed focal SAH in the bilateral precentral sulci (Figure 2). Both the initial and follow-up CT angiograms after 10 days revealed no abnormalities. Symptomatic treatment led to resolution within a month.

**Figure 3:**
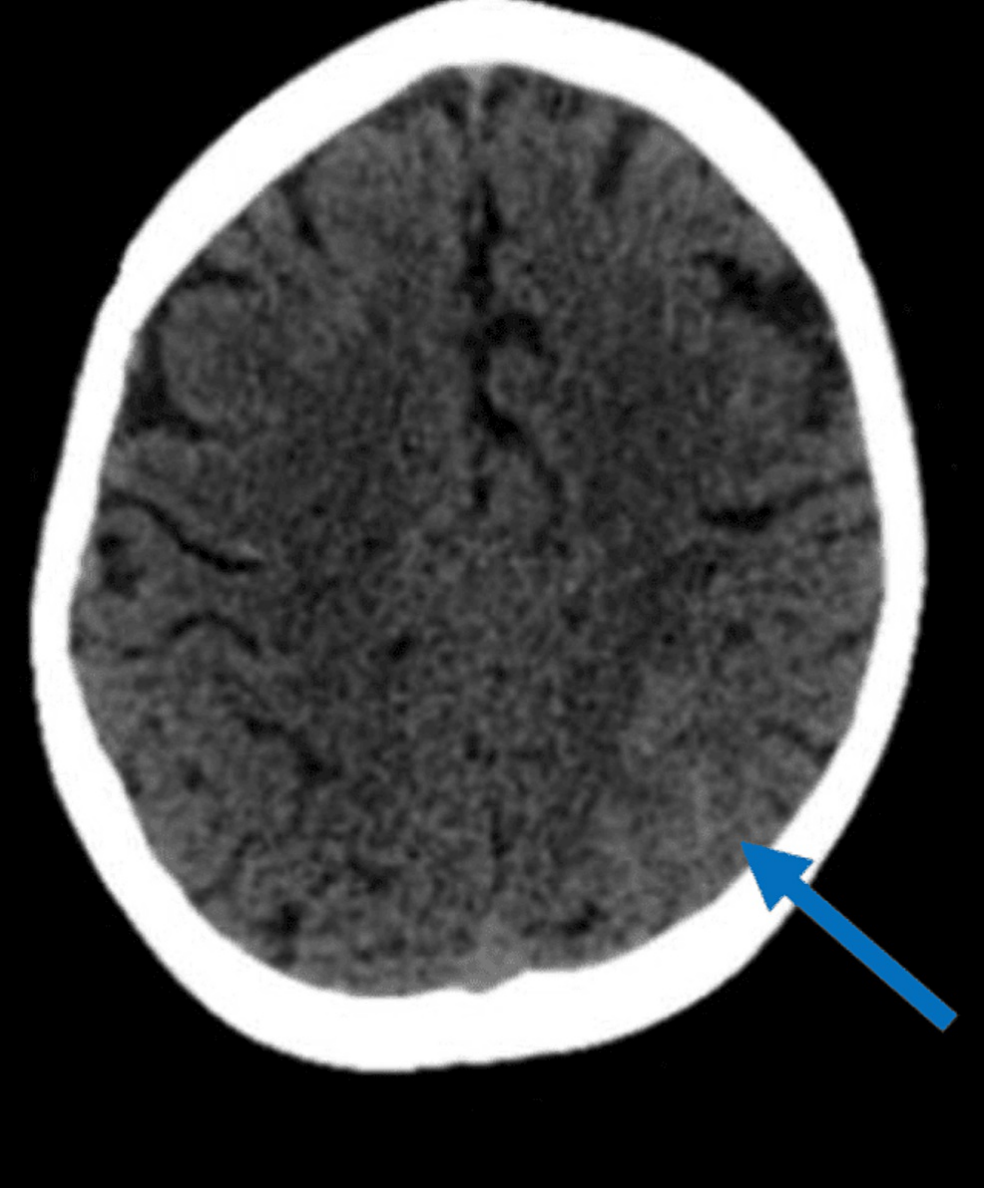
A 45-year-old woman with sudden, severe occipital headache, photophobia, and nausea without any focal neurological deficits. Unenhanced CT shows focal SAH in the left posterior parietal region (blue arrow). CT angiogram (not shown) was normal. CT: computed tomography; SAH: subarachnoid hemorrhage

Additionally, a 45-year-old woman reported a sudden onset of severe occipital headache, photophobia, and nausea without focal neurological deficit or triggering factors. The initial CT scan showed focal SAH in the left posterior parietal region, while the CT angiogram was initially normal (Figure 3). A repeat CT angiogram after 10 days revealed no focal lesions. The patient's symptoms improved within three weeks with symptomatic treatment, and no new symptoms emerged.

These cases highlight the variability in initial imaging presentations of RCVS, ranging from normal findings to focal SAH with a normal angiogram. Identifying vasoconstriction in distal branches on an angiogram poses a diagnostic challenge in RCVS [5].

Cases 4 and 5

**Figure 4:**
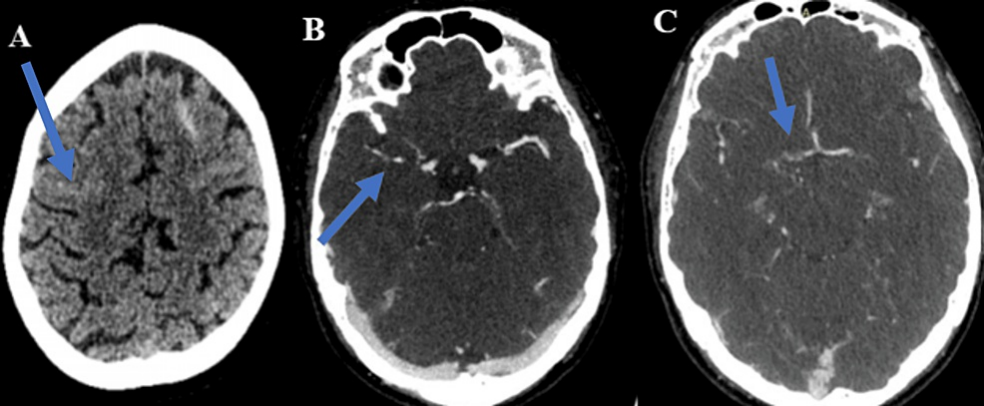
A 52-year-old woman with sudden onset of severe headache. (A) Unenhanced CT shows focal SAH in both frontal regions (right side - blue arrow). (B-C) CT angiogram shows narrowing (blue arrows) in the right middle cerebral artery (B) and right A1 segment of the anterior cerebral artery (C). Follow-up CT angiogram after five months (not shown) shows resolution of the narrowing. CT: computed tomography; SAH: subarachnoid hemorrhage

A 52-year-old woman reported a sudden onset of severe headaches without any identifiable triggering agents. She had no history of hypertension or diabetes, and her blood pressure was within the normal range during physical examination. The initial CT scan revealed SAH in the bilateral frontal regions (Figure 4), and the CT angiogram showed focal narrowing in the right middle cerebral artery and the A1 segment of the anterior cerebral artery on the right side.

**Figure 5:**
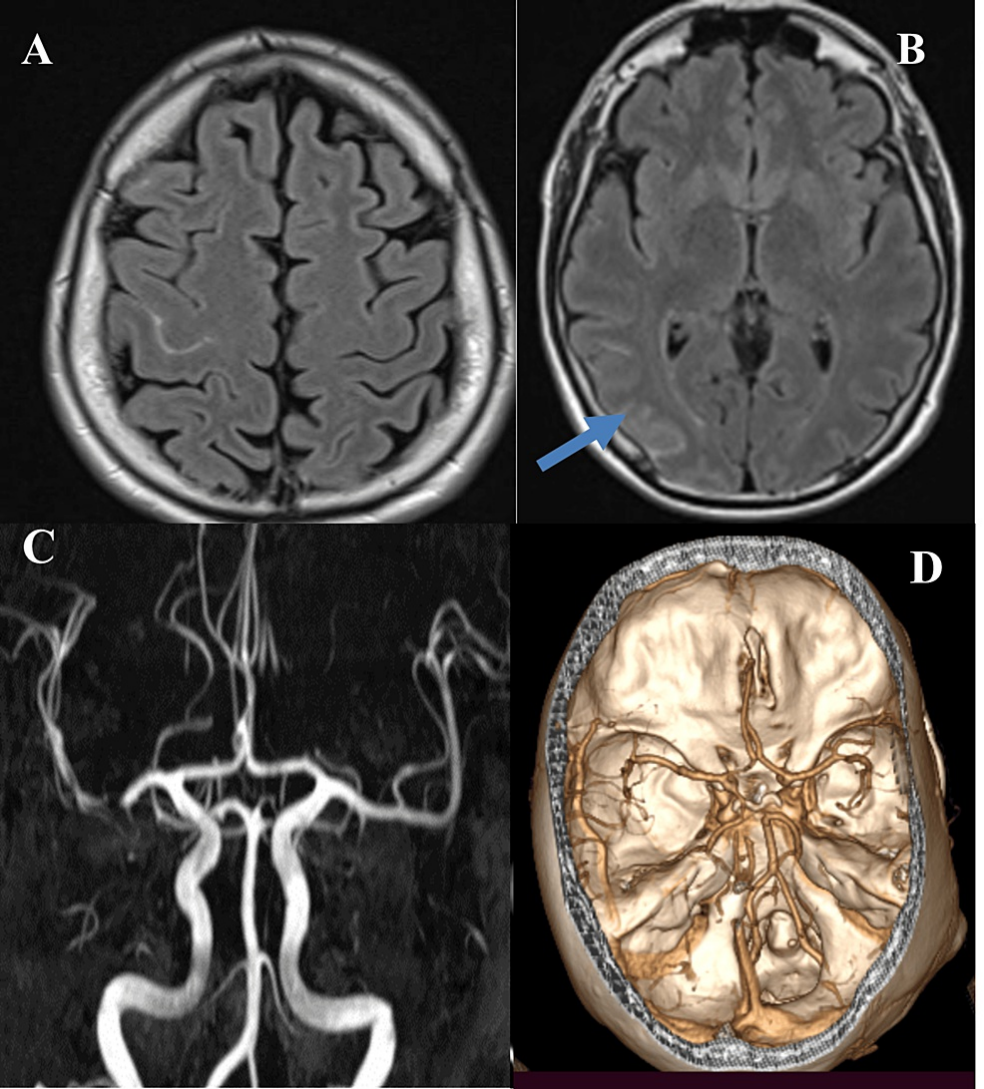
A 48-year-old man with sudden onset of left-sided incoordination and left-sided facial numbness intermittently over two weeks. The initial non-contrast CT (not shown) revealed focal SAH in the right central sulcus. The CT angiogram (not shown) was initially normal. (A-B) FLAIR MRI shows focal SAH in the right central sulcus and the parietooccipital region (blue arrow). (C) MRI angiogram demonstrated focal stenosis in the right middle cerebral artery. (D) Follow-up 3D CT angiogram after six months shows resolution of the right middle cerebral artery stenosis. CT: computed tomography; SAH: subarachnoid hemorrhage; FLAIR: fluid-attenuated inversion recovery; MRI: magnetic resonance imaging

Similarly, a 48-year-old man (Figure 5) experienced a sudden onset of left-sided incoordination and left-sided facial numbness intermittently over two weeks. There was no history of hypertension or diabetes, and he was not on any regular medication. Despite being normotensive during examination, the initial non-contrast CT study revealed focal SAH in the right central sulcus. The CT angiogram was initially normal, but subsequent MRI disclosed SAH in the right central sulcus, occipital, and parietal sulci. The MRI angiogram demonstrated focal stenosis in the right middle cerebral artery. A conventional angiogram confirmed focal stenosis in the right middle cerebral artery.

In both cases, CSF analysis, including protein and white blood cell (WBC) levels, fell within the normal range. Vasculitis workup revealed normal results, leading to the diagnosis of RCVS. Both patients' symptoms improved over three months with the administration of nimodipine, a calcium channel blocker.

These cases underscore the variability in RCVS presentations, emphasizing that RCVS can manifest with or without the classic symptom of thunderclap headache. Radiological findings may overlap with vasculitis, but a normal CSF analysis and a positive response to calcium channel blockers proved crucial in differentiating RCVS from primary angiitis of the central nervous system (PACNS).

Case 6

A 39-year-old woman experienced her inaugural seizure episode, presenting without any reported focal neurological deficits. The initial CT scan showed SAH in the right central sulcus with a normal CT angiogram. A subsequent CT angiogram, performed two weeks later, showed no focal abnormalities. After this incident, no additional seizure episodes were reported.

This case emphasizes that RCVS can manifest with atypical symptoms and that high-level suspicion is important, particularly when focal SAH is present, and after ruling out other potential causes of SAH.

## Discussion

This case series comprehensively explores the diverse imaging presentations and clinical manifestations of RCVS. The cohort of patients revealed a dichotomy, with three individuals displaying the classical thunderclap headache while the remaining three presented with atypical neurological symptoms. These included left-sided facial and hand numbness, left-sided incoordination combined with left facial numbness, and a seizure episode. Three patients exhibited the classical presentation of thunderclap headache [6], while the remaining three presented with other neurological manifestations devoid of headache.

The etiology of RCVS was undetermined in all cases, as none of the patients had a history of precipitating factors [7,8]. Interestingly, while RCVS is typically more common in females, our cohort showed an equal distribution of male and female patients. The ages of patients in our cohort ranged from 39 to 60 years, with an equal distribution of male and female patients. Moreover, it's important to note that this is a very small case series, and these findings cannot be generalized.

In one patient, the initial diagnostic CT imaging, including the MR angiogram, appeared normal. This finding, suggested by Ducros and Bousser [7] may be attributed to vasoconstriction in RCVS occurring initially in the small peripheral vessels before extending to major vessels, thereby remaining undetectable on early imaging.

For the remaining five patients, the initial CT imaging revealed focal SAH primarily confined to cerebral convexities, consistent with literature reports [9]. In one case, the SAH was subtle and might have gone unnoticed if not for the presentation of a severe headache. This distribution of SAH aids in differentiating RCVS from other causes, particularly aneurysmal, which often involves basal cisterns [10].

In our series, three patients exhibited the classic symptom of a thunderclap headache, a sudden-onset severe headache typically described as reaching maximum intensity within seconds to minutes; this is considered a hallmark feature of RCVS often associated with other neurological symptoms [11]. However, a significant subset of our patients presented with other neurological manifestations unaccompanied by the classical thunderclap headache, posing a diagnostic challenge as clinicians may not readily associate these diverse symptoms with RCVS.

Recognizing classical and atypical presentations is crucial for accurate and timely diagnosis. The absence of thunderclap headaches in some cases might lead to initial misdiagnosis or delayed recognition of RCVS, potentially impacting patient outcomes. Clinicians should maintain a high index of suspicion for RCVS, especially when encountering patients with unexplained neurological symptoms, even in the absence of the classic headache [12].

Neuroimaging findings in RCVS, as reported in the literature, encompass intraparenchymal hemorrhage, subdural hemorrhage, focal SAH, ischemic strokes in watershed zones, and cerebral edema [11]. Two patients in our series exhibited focal narrowing and irregularities in the middle cerebral and anterior cerebral arteries on CT/MRI angiograms. It is noteworthy that these angiographic findings overlap with other vasculitis conditions, especially PACNS, underscoring the importance of meticulous differentiation.

According to existing literature, RCVS patients typically present with acute severe headaches, normal MRI brain imaging, or focal SAH, intracerebral hemorrhage, watershed territory infarcts, cerebral edema, and normal CSF analysis [7, 8]. In contrast, PACNS patients present with a gradual onset of symptoms that progress over time. MRI findings often include infarcts and white matter abnormalities, while CSF analysis reveals pleocytosis. The absence of systemic involvement, normal laboratory investigations for vasculitis, and a normal CSF analysis, coupled with focal convexity SAH, aid in differentiating RCVS from other forms of vasculitis in the two patients described in our case series.

Some cases of RCVS are associated with the use of vasoactive substances. Medications like selective serotonin receptor agonists, decongestants, and certain recreational drugs have been implicated. Understanding the potential vasoactive properties of these substances provides insights into their role in triggering vasoconstriction events [7, 8].

The postpartum period is recognized as a predisposing time for RCVS, with cases often occurring in the weeks following childbirth. Hormonal fluctuations, particularly in estrogen levels, during pregnancy and the postpartum period are believed to contribute. The precise mechanisms linking hormonal changes to RCVS development remain an active area of research [7].

A significant challenge lies in cases where RCVS appears to occur spontaneously, without any identifiable precipitating factors. This phenomenon suggests that there may be intrinsic factors, possibly related to vascular reactivity or genetic predispositions, contributing to the development of RCVS. Research efforts are ongoing to unravel the mechanisms behind the spontaneous occurrence of RCVS [8].

Identifying specific causative agents for RCVS poses substantial challenges. The variable and often delayed onset of symptoms, coupled with the absence of a consistent trigger in a significant number of cases, complicates the establishment of direct causal relationships. Additionally, the transient nature of vasoconstriction events and the absence of detectable abnormalities in many routine diagnostic tests further contribute to diagnostic challenges.

In RCVS, the hemorrhage presents as focal and often follows the contours of the cortical sulci, with a predilection for the brain's convexities. This pattern contrasts with ruptured aneurysmal SAH, where the bleeding is typically more diffuse and involves the basal cisterns [13]. Recognizing the distribution of SAH holds clinical significance when evaluating patients with acute neurological symptoms as it directs appropriate management strategies. Aneurysmal SAH often requires urgent neurosurgical intervention to address the underlying vascular pathology, while RCVS is managed differently, focusing on the control of symptoms and addressing potential vasoconstriction through medications such as calcium channel blockers [14].

Other potential differentials for this focal convexity SAH include posterior reversible encephalopathy syndrome (PRES), cerebral amyloid angiopathy (CAA), and cerebral venous thrombosis (CVT). PRES typically manifests as vasogenic edema in the parietal and occipital regions but may also involve the brainstem and basal ganglia. CAA is observed in the elderly and may exhibit cortical-subcortical micro hemorrhage, superficial siderosis, and white matter changes. In CVT, identification is facilitated by a hyperdense thrombosed cortical vein [15, 16].

In some cases of RCVS, there may be intraparenchymal hemorrhage, which can lead to focal neurological deficits depending on the location and extent of the hemorrhage. The presence of intraparenchymal hemorrhage adds a layer of complexity to the diagnosis, as it may mimic other conditions, such as primary intracerebral hemorrhage. Subdural hemorrhage has been reported in association with RCVS. This finding is not as common as SAH in RCVS but contributes to the diverse range of bleeding patterns seen in the syndrome. Subdural hemorrhage may further complicate the clinical presentation and necessitates careful consideration during diagnostic evaluation [14].

Ischemic strokes, resulting from insufficient blood supply to a part of the brain, can occur in the context of RCVS. The vasoconstriction of cerebral arteries may lead to decreased perfusion, causing ischemic events. Ischemic strokes in RCVS can manifest with neurological deficits corresponding to the affected vascular territory. The coexistence of hemorrhagic and ischemic findings adds intricacy to the diagnostic process, requiring a comprehensive evaluation to understand the full extent of vascular involvement. Cerebral edema with PRES-like distribution is another neuroimaging finding reported in RCVS. The mechanisms leading to cerebral edema in RCVS are not fully elucidated but may be related to the changes in vascular tone and permeability. Cerebral edema contributes to the overall complexity of RCVS diagnosis, as it can lead to increased intracranial pressure and worsen clinical outcomes [13].

Angiographic findings, such as focal narrowing and irregularities in cerebral arteries, are hallmark features of RCVS. These changes in vascular caliber are dynamic and reversible, contributing to the syndrome's name. However, the variability in angiographic patterns, including the involvement of different arterial territories, adds complexity to the diagnosis. Careful consideration of angiographic findings is essential for distinguishing RCVS from other vasculopathies, particularly PACNS (Table 1). The temporal evolution of imaging features in RCVS further complicates the diagnostic process. Initial imaging studies may appear normal or show subtle findings, and the characteristic vascular changes may develop over time. The dynamic nature of RCVS necessitates serial imaging studies for a comprehensive assessment, contributing to the intricacies of timely diagnosis [14].

**Table 1:** Reversible cerebral vasoconstriction syndrome vs primary angiitis of the central nervous system

Feature	Reversible Cerebral Vasoconstriction Syndrome	Primary Angiitis of the Central Nervous System [17]
Pathophysiology	Reversible vasoconstriction of cerebral arteries, often triggered by various factors.	Primary inflammatory disorder affecting small- and medium-sized blood vessels in the brain.
Clinical presentation	Sudden, severe thunderclap headache; may present with other neurological manifestations.	Gradual onset of symptoms, including progressive neurological deficits, headaches, and seizures.
Imaging findings	Dynamic and reversible vasoconstriction, subarachnoid hemorrhage; changes over serial studies.	Multifocal stenosis or occlusion of small- to medium-sized vessels; chronic and persistent changes.
Cerebrospinal fluid analysis	Usually unremarkable; no evidence of inflammation or pleocytosis.	May show pleocytosis, elevated protein levels, or evidence of intrathecal immunoglobulin synthesis.
Temporal course	Reversible and often self-limiting course; symptoms and imaging findings improve over days to weeks.	Chronic and progressive course; symptoms may persist or worsen over time without appropriate treatment.
Treatment approach	Supportive care; symptomatic treatment for headaches; calcium channel blockers for vasoconstriction.	Immunosuppressive therapies (e.g., corticosteroids, immunomodulators) to control the underlying inflammatory process.

## Conclusions

RCVS presents a fascinating challenge in neurology, characterized by the reversible constriction of cerebral arteries leading to diverse clinical manifestations. While the classical thunderclap headache is a hallmark, this case series highlights the variability in RCVS presentations, including cases without this typical feature. The absence of a consistent trigger in these cases underscores the enigmatic nature of RCVS etiology, involving transient disturbances in cerebral vascular tone.

Our exploration of six cases, ranging from thunderclap headaches to other neurological manifestations, emphasizes the importance of recognizing the full spectrum of RCVS presentations. The diverse age distribution and equal gender representation further contribute to the heterogeneity of this syndrome. Notably, a high index of suspicion is crucial, as RCVS can manifest with atypical symptoms, requiring careful differentiation from other etiologies. Imaging findings, from normal scans to focal SAHs, reflect the dynamic nature of RCVS, posing diagnostic challenges. The unique distribution of SAH in cerebral convexities proves vital in distinguishing RCVS from aneurysmal causes. Neuroimaging complexities, including intraparenchymal hemorrhage, subdural hemorrhage, ischemic strokes, and cerebral edema, further underscore the intricacies of RCVS diagnosis. In conclusion, this case series contributes valuable insights into the diverse clinical phenotypes, radiological findings, and management considerations associated with RCVS. We aim to enhance the understanding of this intriguing syndrome through meticulous examination and comparison, emphasizing the need for a comprehensive evaluation to capture its varied presentations in clinical practice. However, given the complexity and heterogeneity observed, further investigation through larger studies may be warranted to elucidate the full spectrum of diverse phenotypes.
